# Bromodomain and extraterminal domain inhibitor enhances the antitumor effect of imatinib in gastrointestinal stromal tumours

**DOI:** 10.1111/jcmm.14945

**Published:** 2020-01-19

**Authors:** Jianfeng Mu, Pengfei Sun, Zhiming Ma, Pengda Sun

**Affiliations:** ^1^ Department of Gastric and Colorectal Surgery The First Hospital of Jilin University Changchun China; ^2^ Changchun Railway Medical Insurance Management Office Changchun China; ^3^ Department of Gastrointestinal Nutrition and Hernia Surgery The second hospital of Jilin University Changchun China

**Keywords:** bromodomain and extraterminal domain, BRD4, gastrointestinal stromal tumour, imatinib resistance, JQ1

## Abstract

In gastrointestinal stromal tumours (GISTs), the function of bromodomain‐containing 4 (BRD4) remains underexplored. BRD4 mRNA abundance was quantified in GISTs. In the current study, we investigated the role of BRD4 in GISTs. Our results show a significant enhancement in BRD4 mRNA and a shift from very low‐risk/low‐risk to high‐risk levels as per NCCN specifications. Overexpression of BRD4 correlated with unfavourable genotype, nongastric location, enhanced risk and decreased disease‐free survival, which were predicted independently. Knockout of BRD4 in vitro suppressed KIT expression, which led to inactivation of the KIT/PI3K/AKT/mTOR pathway, impeded migration and cell growth and made the resistant GIST cells sensitive to imatinib. The expression of KIT was repressed by a BRD4 inhibitor JQ1, which also induced myristoylated‐AKT‐suppressible caspases 3 and 9 activities, induced LC3‐II, exhibited dose‐dependent therapeutic synergy with imatinib and attenuated the activation of the PI3K/AKT/mTOR pathway. In comparison with their single therapy, the combination of JQ1/imatinib more efficiently suppressed the growth of xenografts and exhibited a reduction in KIT phosphorylation, a decrease in Ki‐67 and in the levels of phosphorylated PI3K/AKT/mTOR and enhanced TUNEL staining. Thus, we characterized the biological, prognostic and therapeutic implications of overexpressed BRD4 in GIST and observed that JQ1 suppresses KIT transactivation and nullifies the activation of PI3K/AKT/mTOR, providing a potential strategy for treating imatinib‐resistant GIST through dual blockade of KIT and BRD4.

## 1 INTRODUCTION

The majority of GISTs (gastrointestinal stromal tumours) possess mutually exclusive mutations in platelet‐derived growth factor subunit A (PDGFRA) or KIT, which constitutively activate the encoded receptor tyrosine kinases (RTKs) to control the response to imatinib treatment and lead to tumorigenesis.^1^, ^2^ In adults, 10%‐15% of GISTs exhibit wild‐type KIT and PDGFA genes but may bear mutated neurofibromatosis type 1 (NF1), BRAF, or succinate dehydrogenase (SDH) genes.^3^, ^4^ Both the NIH (Bethesda, MD) and National Comprehensive Cancer Network (NCCN) schemes are deemed effective for risk stratification in GIST, whereas their prognostic utility is not uniformly validated, and the NCCN scheme lacks sufficient evidence‐based data for some uncommon settings.^5^, ^6^ Another challenge in the current care of GIST patients is the inevitable resistance to imatinib that emerges following initial responses due to acquired secondary mutations.^7^, ^8^ Therefore, it is desirable to characterize aberrations in nonkinase biochemical pathways that may crosstalk with or regulate RTK‐driven signalling to overcome the current limitations in prognosis and drug resistance, particularly for cases refractory to imatinib.^9^, ^10^

In recent decades, large breakthroughs have led to functional evaluation, epigenetic profiling and targeted therapy in human cancer.^11^ Cancer cells characteristically exhibit aberrant epigenetic regulation and use the regulatory components of the chromatin to carry out transcriptional programmes initiating oncogenesis.^12^, ^13^ There are two particularly important epigenetic readers, bromodomain and extraterminal domain (BET) proteins, that have two characteristic tandem bromodomains (BD1 and BD2) and primarily recognize the acetylated lysine of histones H3 and H4, and occasionally acetylated nonhistone proteins, to modulate gene expression.^11^, ^14^, ^15^ A well‐studied BET family member, BRD4, usually recruits the P‐TEFb transcription elongation factor or other transcriptional factors or chooses modifiers of histones to promote activation of target genes at the transcriptional stage.^16^, ^17^ BRD4 is crucially involved in several cellular processes, including the progression of the cell cycle, cell growth control, apoptosis and tumour initiation.^18^ BRD4 is often overexpressed and clinically associated with various human cancers via its elevation of the expression and enhancement of the oncogenic functions of major proteins in cancer, such as aldehyde dehydrogenases (ALDH) in ovarian cancer, androgen receptor (AR) and ETS‐related gene (ERG) in prostate cancer and c‐Myc in leukaemia.^19^, ^20^ BRD4 was shown to regulate PTEN and the PI3K/AKT pathway.^21^, ^22^, ^23^ The important roles of BRD4 in transcriptional activation and the initiation of tumour formation have led to the consideration of selective inhibition of BRD4 as a promising therapeutic strategy against cancer.^24^ The amino‐terminal bromodomains of BRD4 have been targeted using several selective small‐molecule inhibitors, such as I‐BET762 and JQ1, and promising antiproliferative effects have been observed along with promising clinical significance in a range of cancers.^25^, ^26^ The first‐in class thienodiazepine small‐molecule JQ1 binds to BET protein bromodomains with acute complementarity and an affinity in nanomoles, resulting in transient, competitive and robust displacement of BRD4 from acetylated chromatin, thus selectively occluding transactivation of specific gene sets in a context, or dose‐dependent manner.^13^, ^27^ These findings established a convincing logic for targeting BRD4 in cancer therapy.

In this study, the levels of mRNA and protein expression were significantly increased in higher‐risk tumours. Furthermore, protein overexpression was strongly associated with unfavourable KIT/PDGFR/BRAF mutation types and independently predicted poor disease‐free survival (DFS) in primary imatinib‐naïve GISTs. In imatinib‐resistant GIST cells, stable silencing of endogenous BRD4 decreased cell growth and migration and enabled resensitization to imatinib, concomitant with significant suppression of the activity and/or expression of kinases in the PTEN/PI3K/AKT/mTOR signalling cascade. Apart from inducing apoptosis and autophagy, BRD4 inhibition using JQ1 apparently inhibited total and phosphorylated KIT levels through transcriptional repression and inactivated downstream kinases. Compared with either drug alone, the therapeutic synergy of combined therapy with JQ1 and imatinib was substantiated in vitro and in vivo, with concomitant downregulation of KIT and the phosphorylation levels of proteins in the PTEN/PI3K/AKT/mTOR pathway. Taken together, we robustly characterized BRD4 as a progression‐associated metabolic driver for accurate prognostication in GIST and provided a rationale for dual blockade of KIT and BRD4 in GIST therapies.

## 2 MATERIALS AND METHODS

### 2.1 Cell lines

After imatinib therapy, GIST48 cells harbouring a homozygous V560D mutation acquired a heterozygous D820A mutation in KIT exon 17, and GIST430 cells harbouring a heterozygous in‐frame deletion in KIT exon 11 developed a heterozygous missense mutation in exon.^28^ Authentication of both cell lines was performed by short‐tandem‐repeat genotyping, and lines were periodically confirmed to be mycoplasma free using PlasmoTest (Invivogen) and maintained in IMDM (Invitrogen) containing FBS (10%), penicillin/streptomycin (100 U/mL) and l‐glutamine (4 mM; Invitrogen) at 37°C in CO_2_ (5%).

### 2.2 Tumour cohorts

The Institutional Review Boards approved this study. For IHC, we employed constructed tissue microarrays (TMAs) of 300 primary GISTs resected before 2012 with triplicate cores in each case. The Recut TMA sections yielded 300 samples with information regarding the BRD4 immunoexpression status (Table 1), including 177 samples that were successfully genotyped for KIT/PDGFRA/BRAF. Independent cases of GIST with available frozen samples were used to determine BRD4 mRNA abundance by real‐time PCR. All cases evaluated for immunoexpression and mRNA abundance were unexposed to imatinib prior to relapse of the disease and unrelated to neurofibromatosis type I.

**Table 1:** Associations of BRD4 level with clinicopathologic parameters in GIST patients

	BRD4 expression		*P*
	Low level	High level	
Gender			>.05
Male	109	43	
Female	106	42	
Age (y)	56.3 ± 14.33	58.4 ± 12.23	>.05
Location			>.05
Gastric	114	46	
Nongastric	101	39	
Tumour size (cm)	5.2 ± 2.34	6.5 ± 3.5	<.01
NIH risk			<.001
Low/very low	92	17	
Intermediate	73	20	
High	50	48	
NCCN guideline			<.001
None/very low	72	4	
Low	69	19	
Moderate	36	14	
High	38	48	

### 2.3 IHC

For antigen retrieval, TMA sections were microwaved and incubated with a primary antibody against BRD4 (BD transduction), and the protein expression was detected using a ChemMate EnVision Kit from Dako. BRD4 immunoexpression was recorded as the mean percentage of labelled cells in the tumoural cytoplasm, and samples with 50% or more tumour cells whose cytoplasms stained moderately or strongly were classified as overexpressing samples.

### 2.4 Myristoylated‐AKT transfection

The lentiviral vectors harbouring constitutively active, myristoylated AKT were purchased from Addgene.

### 2.5 BRD4 knockout

BRD4 sgRNA Crispr Lentivirus was got form abm (sgRNA#1: AGATTTCTCAATCTCGTCCC/sgRNA#2: ACTAGCATGTCTGCGGAGAG). GIST48 and GIST430 cells were used to generate BRD4 knockout cells. For the selection, lentivirus infected cells were treated with puromycin (5 μg/mL) after 1 day of infection and incubated at 37°C. Western blotting was used to confirm the knockout cells.

### 2.6 In vitro wound‐healing assays

Wound‐healing studies were carried out as described previously.^10^ Briefly, using the tip of a P‐100 pipeteman, slashes were made in nearly confluent cell cultures after the inhibitors were added. Photographs of the plates were taken using an inverted Leica DMI 3000B microscope (Leica Microsystems). Experiments were performed in triplicate.

### 2.7 Colony formation assay

Single‐cell suspensions (10 mL) were seeded in p100 culture dishes in triplicate. After 24 hours, imatinib, JQ1, singly, a combination, or DMSO, was added to the cell culture and incubated further for 10 days. Likewise, MK‐2206, an AKT inhibitor, was used as a single agent or in combination with imatinib. Finally, at the end of the experiment, the cells were fixed and stained for 20 minutes with crystal violet (0.5%) and then washed, dried and photographed.

### 2.8 RNA extraction and real‐time quantitative PCR

Real‐time PCR was performed as previously described.^29^, ^30^ Total RNA was extracted from GIST cells using TRIzol reagent or phenol‐chloroform. For the latter process, RNA was precipitated with ethanol and resuspended in water treated with diethyl pyrocarbonate. The total RNA was converted into cDNA using the Moloney murine leukaemia virus reverse transcriptase kit. Template cDNA (1 µL) was used for the real‐time qPCR, containing 5× HS SYBR qPCR forward and reverse primers (10 mmol/L each) for experimental or control genes, and the reaction was carried out on a CFX96 Real‐Time Detection System from BioRad according to the manufacturer's protocol. Simultaneously, each sample was processed to determine *GAPDH* (glyceraldehyde‐3‐phosphate dehydrogenase) levels, which is a housekeeping gene and was used as a control gene in these experiments, and the absolute mRNA levels of each gene of interest were normalized relative to the GAPDH level. Quantitative data were generated based on the number of cycles needed for the fluorescence generated by amplification to achieve a specific detection threshold (the Ct value). The primers are list as followed: BRD4: forward primer, 5′‐ACCTCCAACCCTAACAAGCC‐3′ and reverse primer, 5′‐TTTCCATAGTGTCTTGAGCACC‐3′; cKIT: forward primer, 5′‐GTCTCCTCTGACTTCAACAGCG‐3′ and reverse primer, 5′‐ACCACCCTGTTGCTGTAGCCAA‐3′; GAPDH: forward primer, 5′‐ ACATCGCCAGAGCCAACG‐3′ and reverse primer, 5′‐ ATCCACTTTAATTTCGGGTCAA‐3′.

### 2.9 Western blot assay

Western blot assay was performed as previously described.^31^, ^32^ Total cell protein was isolated using NP40 lysates in the presence of inhibitors of proteases and phosphatases. The proteins were separated by SDS‐PAGE and transferred to PVDF membranes. Blots were incubated overnight with primary antibodies at 4°C. Then, blots were incubated with the corresponding secondary antibodies conjugated with HRP. Immunoreactive bands were examined with the ECL reagent from Thermo Scientific. The primary antibodies are list as followed: BRD4 (#13440), cleaved caspase 3 (#9661), cleaved caspase 9 (#9509), LC3 I/II (#4108), p‐PI3K (#4228), PI3K (#4255), p‐AKT (#4060), AKT (#9272), p‐mTOR (#2976), mTOR (#2972), p‐cKIT (#3391), cKIT (#3074, Cell Signaling Technology) and β‐actin (#5441, Sigma).

### 2.10 GIST xenograft models

To generate xenografts of subcutaneous human tumours, the flanks of 5‐ to 8‐week‐old female nu/nu mice were s.c. inoculated with a suspension of 1 × 10^7^ GIST430 cells/mL (in 100 µL) in Dulbecco's phosphate‐buffered saline. The Committee for Ethics of Animal Experimentation approved the animal experimental protocols, and the experiments were conducted as per the Guidelines for Animal Experiments of The Second Hospital of Jilin University. After each subcutaneous tumour reached a volume of ~100 mm^3^, the mice were arbitrarily categorized into treatment groups and drug was administrated. Mice were given daily oral administration of 50 µL of vehicle (negative control), imatinib (30 mg/kg), JQ1 (30 mg/kg) or the drug combinations indicated above. The animals were randomly categorized into 4 groups for each treatment regimen, as indicated above. The volume and weight of tumours and the general health of the mice were recorded. The mice were then sacrificed, and tumours were excised and histopathologically examined. For staining, FFPE (formalin‐fixed, paraffin‐embedded) tissues were sectioned into 4‐µm sections.

### 2.11 Statistics

To present all quantitative data, the mean ± standard deviation (SD) was used from at least three independent experimental data points. Prism V (GraphPad Software) was used for statistical analyses. One‐way analysis of variance (ANOVA) or an unpaired two‐tailed Student's *t* test was applied to determine the statistical differences between two groups of data. When *P* < .05, the data were deemed significant.

## 3 RESULTS

### 3.1 Overexpression of BRD4 mRNA and protein correlated with unfavourable and poor clinicopathologic outcomes

**Figure 1:**
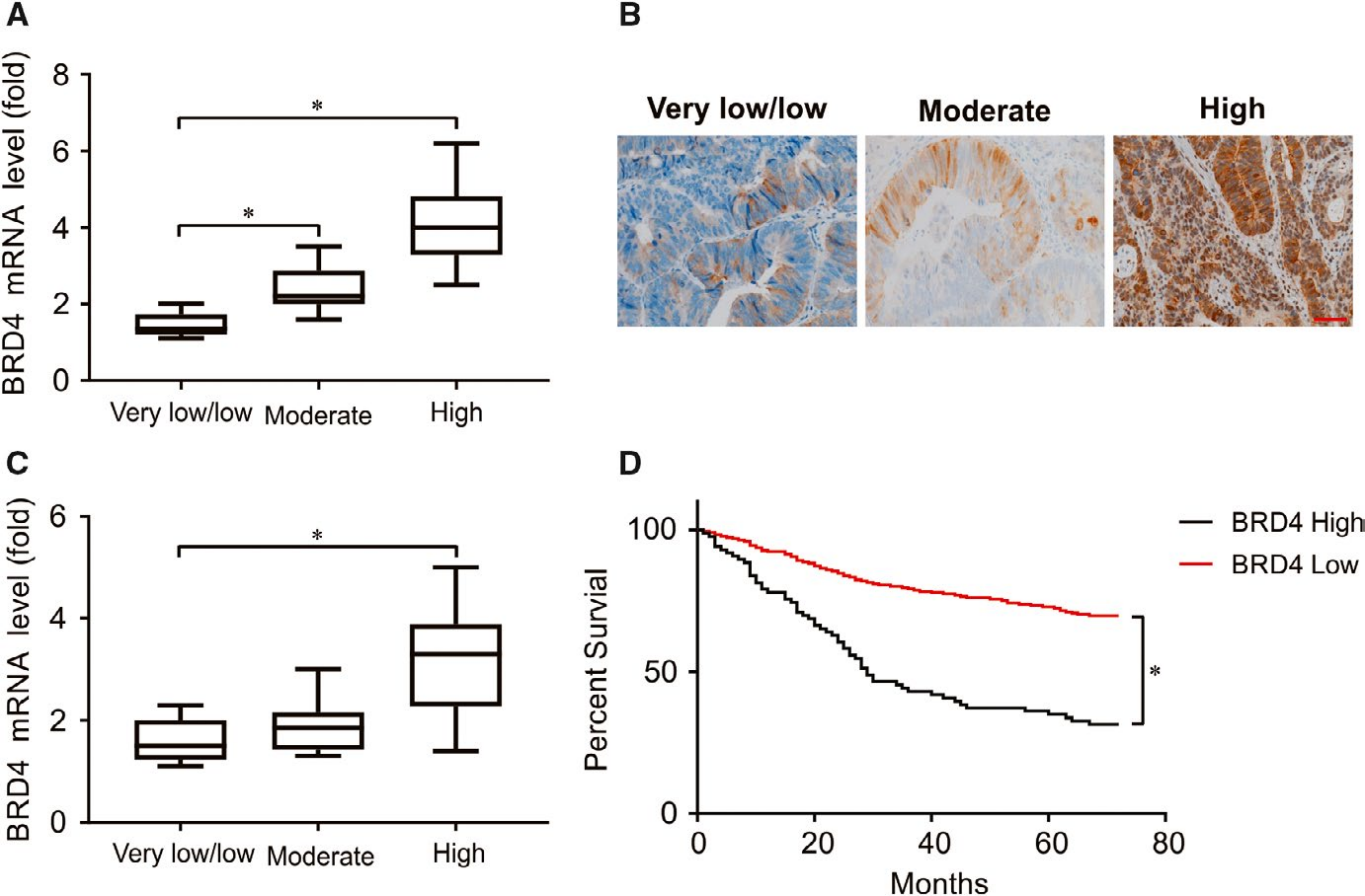
The clinical relevance of increased BRD4 mRNA and protein expression in GIST. A, Real‐time PCR of GIST demonstrated significantly increased BRD4 mRNA abundance in higher risk categories using NCCN guidelines. B, The level of BRD4 in representative low‐, moderate‐ and high‐risk GIST was analysed by IHC staining. C, Real‐time PCR of GIST demonstrated significantly increased BRD4 mRNA abundance in higher risk categories using NCI guidelines. D, In log‐rank analyses, BRD4 overexpression was highly predictive of poor disease specific survival. *P < .05

The endogenous BRD4 mRNA levels in the LCM‐isolated tumour cells of 40 primary GISTs were quantified, revealing significant upregulation in higher risk categories defined by both the NCCN guidelines (Figure 1A,B) and the NIH scheme (Figure 1C). In regard to BRD4 mRNA levels, NCCN guidelines more effectively distinguished GISTs of various risk categories. In contrast, no significant difference was observed between low‐risk and intermediate‐risk cases using the NIH scheme. Consistent with the frequent BRD4 transcriptional upregulation in carcinomas, BRD4 overexpression in the progression of GISTs likely occurs in part because the transcriptional machinery follows altered signalling stimuli.

To examine the translatability of upregulated mRNA, the clinical relevance of overexpressed protein was analysed in a large set of primary imatinib‐naive GISTs. There were 300 GISTs exhibiting BRD4 immunoexpression (Table 1) with clinical follow‐up data, which comprised 76 no‐ or very low‐risk cases, 88 low‐risk cases, 50 moderate‐risk cases and 86 high‐risk cases based on NCCN guidelines or 109 very‐low/low‐risk cases, 93 intermediate‐risk cases and 98 high‐risk cases per the NIH risk scheme. Importantly, high BRD4 expression was associated with poor overall survival (OS) in GIST patients (Figure 1C). Together, BRD4 overexpression remained an independent adverse prognostic factor, along with increased NIH risk levels and epithelioid histology.

### 3.2 BRD4 knockout attenuated cell growth, migration and PI3K/mTOR/AKT activation

**Figure 2:**
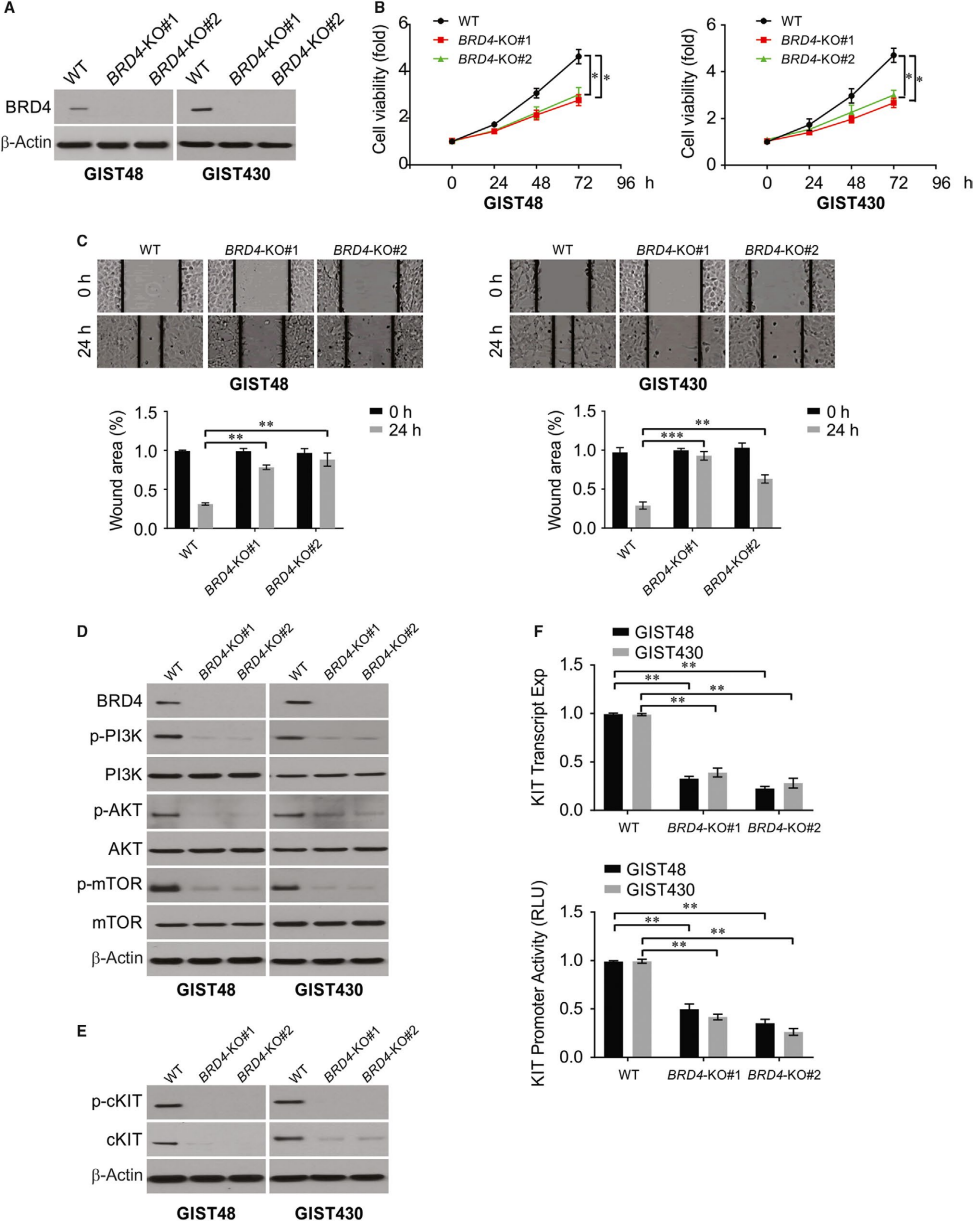
BRD4 knockout attenuated cell migration, imatinib resistance and PI3K/AKT/mTOR signalling activity. A, GIST48 and GIST430 *BRD4*‐KO cell lines were analysed by Western blotting. B, The cell viability was detected in WT and *BRD4*‐KO cells. C, The percentages of wound closure were analysed in WT and *BRD4*‐KO GIST48 and GIST430 cell lines at indicated time points. D, Indicated protein level was analysed by Western blotting in WT and *BRD4*‐KO GIST48 and GIST430 cell lines. E, Indicated protein level was analysed by Western blotting in WT and *BRD4*‐KO GIST48 and GIST430 cell lines. F, KIT transcript and KIT promoter activity were analysed in WT and *BRD4*‐KO GIST48 and GIST430 cell lines. *P < .05; **P < .01

Given the challenge of imatinib resistance in GIST, we focused on imatinib‐resistant GIST48 and GIST430 cell lines to characterize the biological implications of BRD4. As both cell lines overexpressed BRD4, Crispr/cas 9 system was used to knockout BRD4 in GIST48 and GIST430 cells. Two BRD4 knockout (*BRD4*‐KO) clones achieved in these cell lines, which was confirmed by western blotting (Figure 2A). Compared with WT, *BRD4*‐KO cells significantly decreased the number of viable cells in both cell lines (Figure 2B), which is consistent with the known growth‐promoting function of BRD4. Moreover, the wound‐healing assay exhibited significantly fewer migrating cells when BRD4 was depleted, indicating that BRD4 has a promigratory effect (Figure 2C). Accumulating evidence has revealed that the PI3K/AKT/mTOR pathway plays important roles in relaying PTEN signalling in GIST and the neoplastic lipogenesis of other cancers. Therefore, we explored the potential regulatory basis of these kinases linked to the overexpression of BRD4 in GIST cell models, demonstrating that the phosphorylated forms of PI3K, AKT and mTOR were all prominently downregulated by BRD4 knockout (Figure 2D). In addition, the total and phosphorylation of KIT protein was significantly decreased in *BRD4*‐KO GIST48 and GIST430 cell lines (Figure 2E). Furthermore, KIT mRNA expression and promoter activity were found suppressed in *BRD4*‐KO cells as measured by qRT‐PCR (Figure 2F).

### 3.3 The sensitivity of imatinib‐resistant GIST cells is restored by inhibition of BRD4

**Figure 3:**
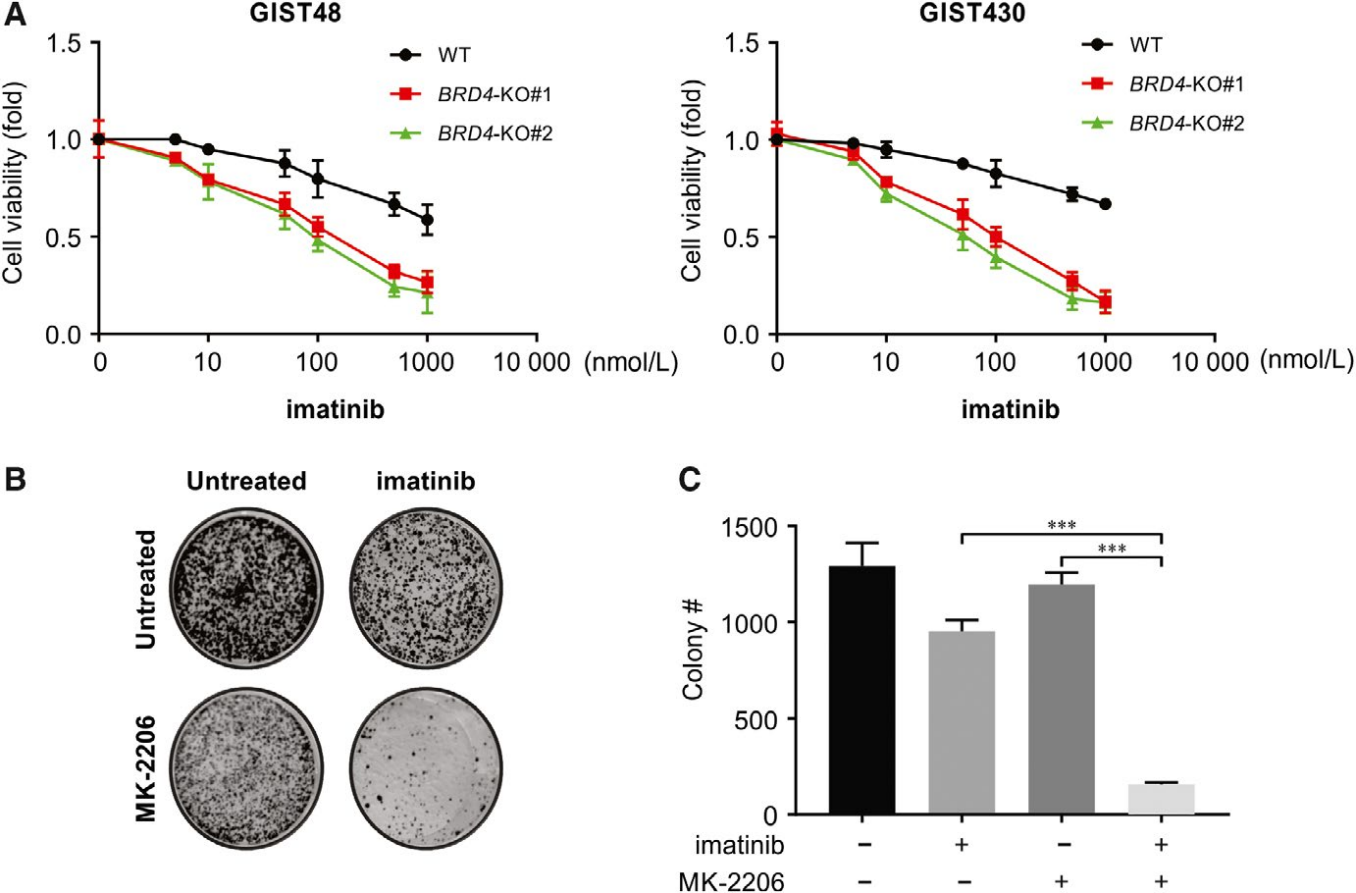
Knockout BRD4 resensitizes GIST48 and GIST430 cells to imatinib. A, WT and *BRD4*‐KO GIST48 and GIST430 cell lines were treated with imatinib, cell viability was analysed by MTS. B, C, Colony formation of GIST48 treated with imatinib, MK‐2206, and their combination. ***P < .001

Next, we investigated whether BRD4 inhibition restores imatinib sensitivity in GIST. Depending on the efficiency of the *BRD4‐*KO clones, BRD4 silencing resulted in a variable but significantly increased percentage of nonviable GIST48 and GIST430 cells after treatment with imatinib (Figure 3A), implying that suppression of BRD4 expression may reduce imatinib resistance.

One of the downstream intracellular signalling cascades of BRD4/PTEN signalling is the AKT/mTOR pathway. Thus, we investigated whether PI3K/AKT pathway inhibition leads to re‐sensitization of imatinib‐resistant GIST to imatinib. For this, GIST48 cells were treated with MK‐2206 (a potent PI3K/AKT inhibitor) as a single agent or in combination with imatinib for 10 days; the long‐term viability of the cells was assessed, and the cells stained with crystal violet. We observed that inhibition of PI3K/AKT reversed the sensitivity of GIST48 cells to imatinib, as revealed by the significant cell growth inhibition seen in GIST48 cells when they were exposed to a combination of imatinib and MK‐2206 (Figure 3B,C). Collectively, these data indicate that the AKT/mTOR signalling pathway plays a vital role in imatinib resistance in GIST48 cells.

### 3.4 JQ1 inhibited viability, exhibited synergy with imatinib and induced apoptosis and autophagy in vitro

**Figure 4:**
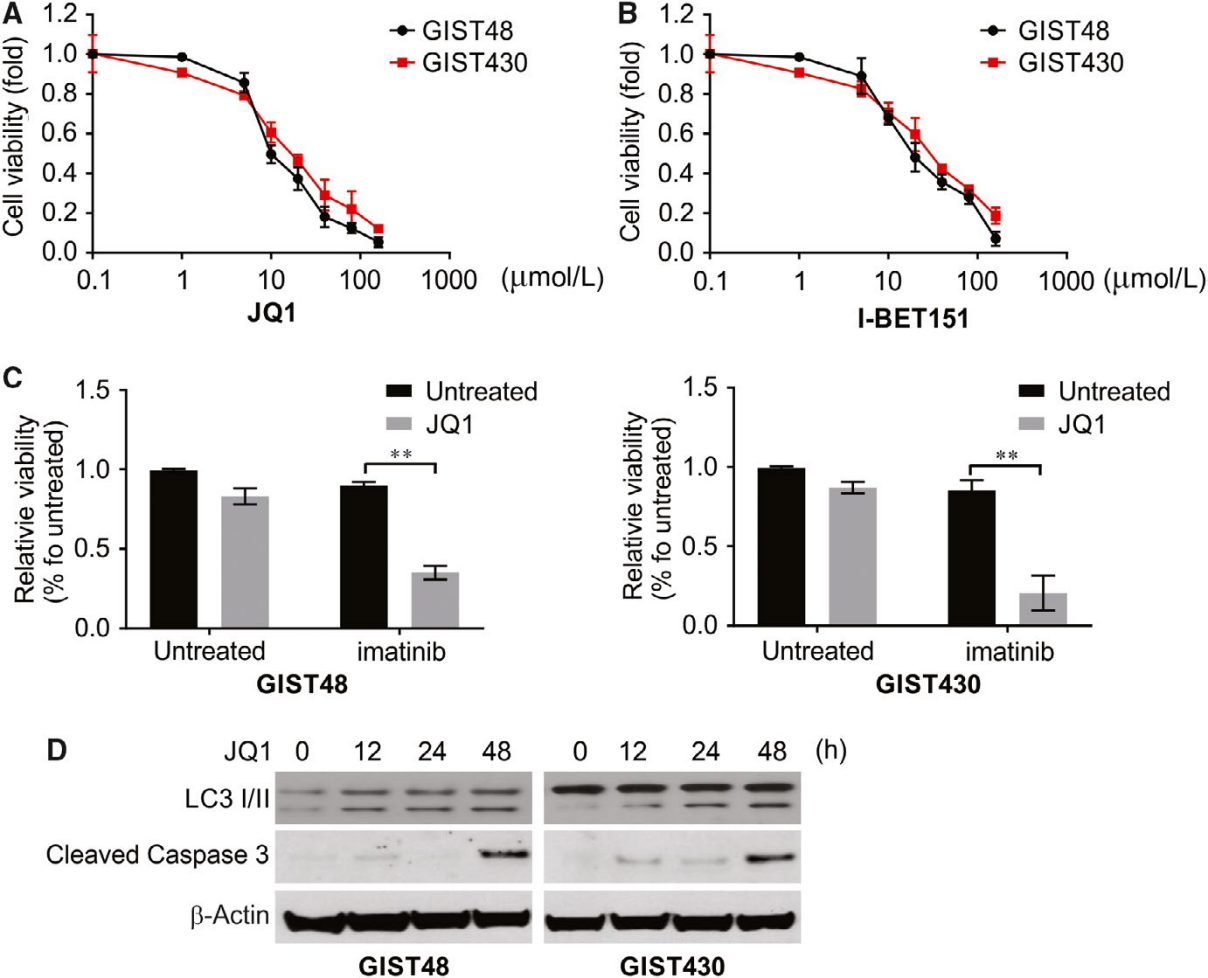
BET inhibitor overcomes imatinib resistance in GIST. A, B, GIST48 and GIST430 cell lines were treated with JQ1 or I‐BET‐151 at indicated concentration. Cell viability was analysed by MTS. C, GIST48 and GIST430 cell lines were treated with imatinib with or without low concentration JQ1 (1 μmol/L). Cell viability was analysed by MTS. D, GIST48 and GIST430 cell lines were treated with 10 μmol/L JQ1 at indicated concentration, indicated protein level was analysed by Western blotting. **, *P* < .01

A synthetic analogue of cerulenin, JQ1, has been shown to potently inhibit BRD4 and suppress the tumour growth of various human cancers in vitro and in vivo.^24^ However, the therapeutic potential and molecular underpinnings of JQ1 remain unexplored in GIST, especially in regard to crosstalk with KIT‐elicited signal transduction. As measured by the MTS assay, JQ1 had a deleterious effect on the survival of both cell lines (Figure 4A). Another BRD4 inhibitor, I‐BET151, also dose‐dependently suppressed the cell viability of both GIST cell lines, although its potency was inferior to that of JQ1 (Figure 4B). Accordingly, we selected JQ1 for further assessment of its potential efficacy in combination with imatinib. The imatinib and JQ1 combination had significantly improved viability‐suppressing capabilities in the GIST48 and GIST430 cell lines (Figure 4C). This finding indicated a synergistic effect between JQ1 and imatinib and the need for validation of the potential of a dual blockade of KIT and BRD4 in vivo. To decipher the mechanism of the JQ1‐induced growth inhibition of imatinib‐resistant GIST cells, we examined whether JQ1 could induce cellular apoptosis, and its ability to do so was validated by significantly induced cleaved caspase‐3 levels in GIST48 and GIST430 cells treated with JQ1 (Figure 4D). In both GIST cell models, JQ1, similar to imatinib, also increased the expression of the LC3‐II isoform, indicating the role of JQ1 in triggering autophagy (Figure 4D). These findings implied an existent but variable synergistic effect of the JQ1/imatinib combination on the induction of cellular autophagy and apoptosis in imatinib‐resistant GIST cells.

### 3.5 JQ1 repressed KIT transactivation and the JQ1/imatinib combination abrogated KIT and PI3K/mTOR/AKT signalling activities more potently than did monotherapies

**Figure 5:**
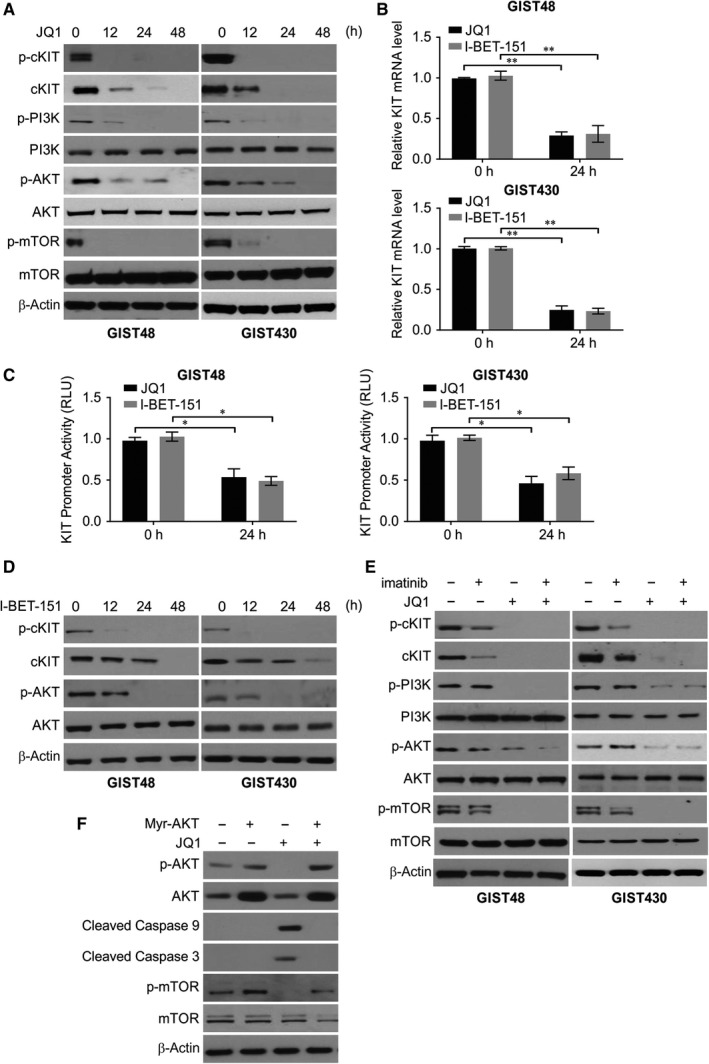
JQ1 regulates PI3K/AKT signalling pathway in GIST. A, GIST48 and GIST430 cell lines were treated with JQ1. Indicated protein level was analysed by Western blotting. B, GIST48 and GIST430 cell lines were transfected with 10 μmol/L JQ1 or 10 μmol/L I‐BET‐151. KIT transcript was analysed. C, GIST48 and GIST430 cell lines were transfected with 10 μmol/L JQ1 or 10 μmol/L I‐BET‐151. KIT promoter activity was analysed. D, GIST48 and GIST430 cell lines were treated with I‐BET‐151. Indicated protein level was analysed by Western blotting. E, GIST48 and GIST430 cell lines were treated with JQ1, imatinib, or their combination. Indicated protein level was analysed by Western blotting. F, GIST430 cell lines transfected with Myr‐AKT were treated with JQ1. Indicated protein level was analysed by Western blotting. *P < .05; **P < .01

Consistent with the knockout results, the phosphorylated KIT level was diminished in JQ1‐treated imatinib‐resistant GIST cells (Figure 5A), which was in line with the significantly decreased KIT mRNA levels (Figure 5B,C). Similarly, I‐BET151 induced significant repression of KIT promoter activity in both cell lines, along with resultant decreases in the levels of mRNA, protein and phosphorylation (Figure 5B‐D). These findings indicate that transcriptional repression is at least partly responsible for the KIT‐repressing effect of pharmacologic inhibition by JQ1, and this repression became more evident when JQ1 was combined with imatinib. Furthermore, treatment with JQ1 or imatinib individually or in combination was examined to determine the effects on KIT phosphorylation and PI3K/AKT/mTOR signalling activity. Consistent with BRD4 silencing, BRD4‐targeting by JQ1 abrogated the activation of PI3K, AKT and mTOR (Figure 5A), with no or relatively slight decreases in the expression of the total forms of individual kinases. Generally, similar to treatment with JQ1, I‐BET151 treatment inhibited the expression and activation of KIT as well as downstream kinases of the AKT signalling cascade in both GIST48 and GIST430 cells (Figure 5D).

Compared with monotherapies or vehicle control, the JQ1/imatinib combination more effectively inactivated the kinases of the AKT/mTOR signalling cascade in GIST430 cells (Figure 5E). Given the known antiapoptotic role of AKT, we transfected cells with myristoylated AKT to elucidate its effect on BRD4 inhibition‐induced apoptosis in GIST430 cells. The GIST430 cells with myristoylated AKT exhibited significantly reduced activities of caspases 3 and 9 triggered by JQ1 (Figure 5F), implying a potential link between AKT inactivation and JQ1‐induced cytotoxicity.

### 3.6 JQ1 monotherapy versus JQ1/imatinib combined therapy in vivo

**Figure 6:**
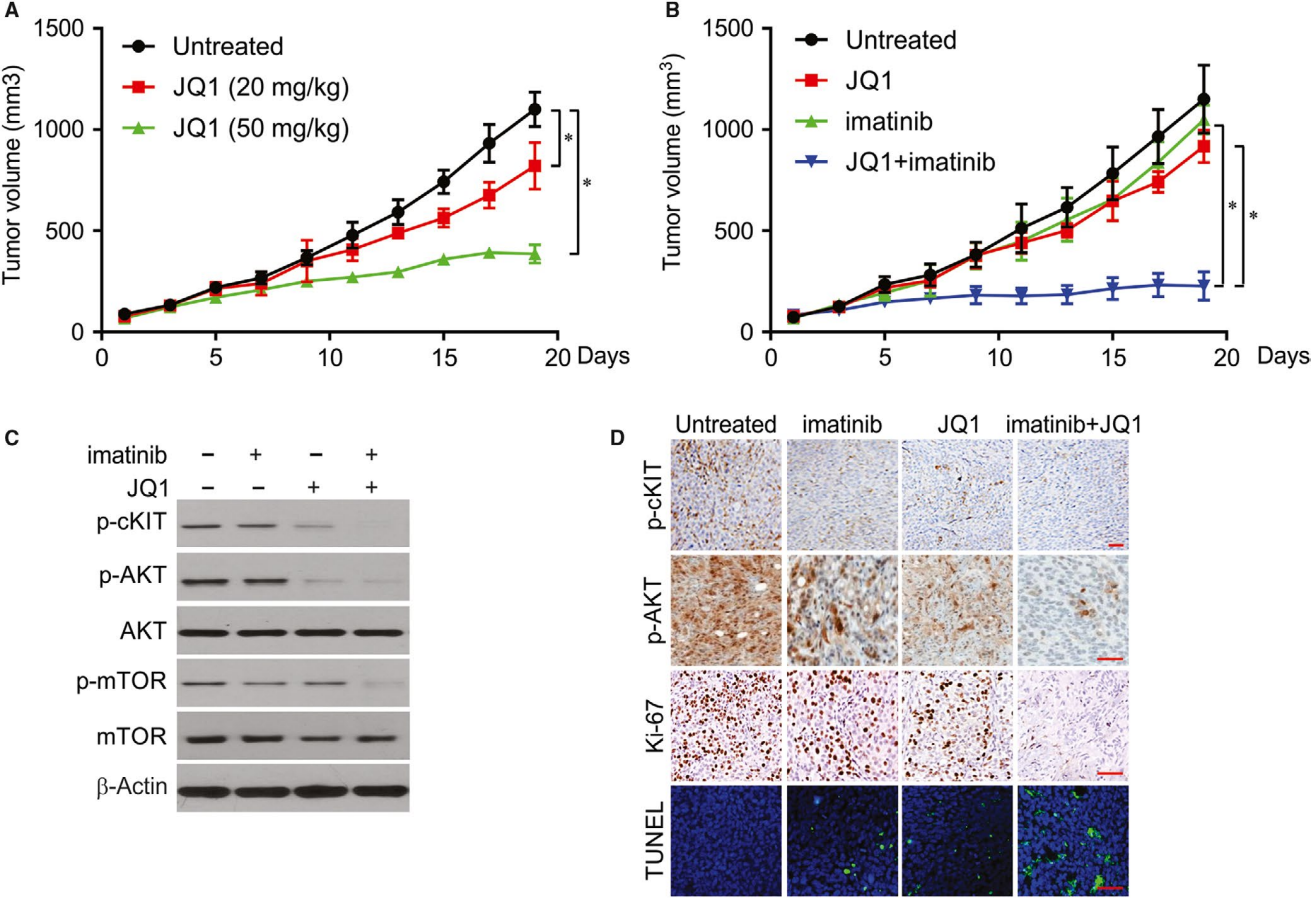
JQ1 enhances the antitumour effect of imatinib in vivo. A, GIST430‐derived xenograft was treated with JQ1 at indicated concentration. Tumour volume at indicated time points after treatment was calculated and plotted with *P* values. B, GIST430‐derived xenograft was treated with imatinib, JQ1, and their combination. Tumour volume at indicated time points after treatment was calculated and plotted with *P* values. C, The levels of indicated protein in randomly selected tumours were analysed by Western blotting. D, The level of indicated protein was analysed by IHC or IF staining. *P < .05

Given the synergy between JQ1 and imatinib, a GIST430‐derived xenograft model was established to analyse the in vivo therapeutic efficacy and antitumour mechanism of JQ1 and JQ1/imatinib combination therapy. The in vivo tumour‐inhibiting effect of JQ1 was significant at both 20 and 50 mg/kg doses compared with that of control group (Figure 6A). Next, we evaluated the therapeutic efficacy in the JQ1/imatinib combination, which from day 19 onwards exhibited more prominent growth‐suppressing capabilities than either JQ1 or imatinib alone (Figure 6B), corroborating the synergy of the combined therapy in vitro. Tumour cells of the control group exhibited diffuse and strong expression of p‐cKIT, p‐AKT and p‐mTOR; a high Ki‐67 proliferative index; and few TUNEL‐positive apoptotic cells. In contrast, the xenografts receiving combined therapy displayed decreases in the levels of p‐cKIT and phosphorylated kinases downstream of the AKT/mTOR pathway (Figure 6C,D). However, these alterations in histomorphology, kinase expression and activity and proliferative and apoptotic markers were mild to moderate in the monotherapy groups receiving imatinib or JQ1 alone.

## 4 DISCUSSION

GISTs mainly possess activating oncogenic ‘driver’ mutations in KIT or sometimes PDGFRA.^33^, ^34^ Imatinib mesylate possibly inhibits the activity of the KIT kinase and is the first‐line drug for unresectable and advanced GIST treatment, attaining at least a limited response in nearly 80% of metastatic patients.^34^, ^35^ Nevertheless, most patients develop resistance to imatinib within 2‐3 years of treatment initiation, making the clinical management of GIST challenging.^36^ Therefore, the identification of new treatment targets is required in GIST to expand the treatment choices for patients who are resistant to small‐molecule tyrosine kinase inhibitors, such as imatinib.^37^ In a variety of GISTs, four chief mechanisms for imatinib resistance have been characterized: (a) acquisition of a secondary point mutation in KIT or PDGFRA; (b) genomic amplification of KIT; (c) alternate kinase activation; and (d) loss of expression of KIT although other mechanisms may contribute to imatinib resistance.^37^, ^38^, ^39^

The BET (bromodomain and extraterminal domain) family of proteins consists of BRD2, BRD3, BRD4 and BRDT, and the bromodomain structure comprises four alpha helices separated by variable loop regions, which can recognize acetylation sites and recruit transcription factors.^13^ Based on its strong effect on transcriptional regulation, the role of the BET family in the promotion of the biological behaviour of cancer cells has been identified.^24^ Furthermore, the BRD4 inhibitor (+)‐JQ1 (JQ1) has been shown to suppress the proliferation of cancer cells, indicating that JQ1 may be a new therapeutic agent for cancer treatment.^25^ However, the clinical application of JQ1 is limited.^25^ Since some cancer cells are insensitive to JQ1 treatment, subsequently leading to treatment failure.^26^, ^40^ Therefore, new drugs or models need to be identified to overcome the obstacles associated with JQ1 treatment.

In this study, GIST tissues exhibited a positive correlation between BRD4 mRNA abundance and risk levels. This result indicated that BRD4 overexpression in GIST is, at least in part, transcriptionally regulated. The strong association between BRD4 overexpression and adverse clinicopathologic variables, such as higher risk levels, was also validated in our cohort of GISTs. Moreover, we confirmed that BRD4 overexpression not only correlated with unfavourable genotypes but also conferred an independent negative prognostic impact, with a twofold higher risk of shorter DFS. A previous study reported that JQ1 inhibited the PI3K/AKT pathway via upregulating PTEN.^41^ The biological implications of overexpressed BRD4 in GIST were supported by our findings that BRD4‐silenced cell lines demonstrated decreased cell growth and migration. Although overexpressed BRD4 has emerged as a potential therapeutic target in various cancer types, knowledge about the biology of BRD4 inhibition in GIST cell and xenograft models is scant.^19^ One such mechanism hypothesized to be involved in imatinib resistance in GIST is the activation of PI3K/AKT signalling.^39^ Indeed, there was an increase in the expression of BRD4 in imatinib‐resistant GIST cells in vitro as well as in the imatinib‐resistant tumour specimens of GIST patients.

In RTK‐targeted therapies, such as imatinib targeting mutant KIT in GIST, acquired resistance frequently follows a short‐lived treatment response.^28^ This predicament urges a fundamental elucidation of the interdependent KIT‐eliciting downstream and collateral pathways essential for sustaining the resistance and survival of tumour cells to develop novel therapeutic strategies.^7^ In GIST cells harbouring refractory mutations, BRD4 knockout indeed inhibited the activity of the PI3K/AKT/mTOR axis and notably increased the susceptibility to imatinib. These results highlighted the therapeutic relevance of overexpressed BRD4 in imatinib‐resistant GIST and the potential of drug combinations for dual blockade of BRD4 and KIT. This is the first time that a study has highlighted the significance of BRD4/PI3K/PTEN/AKT inhibition in surpassing imatinib resistance in GIST, thus indicating that targeting BRD4 may be a promising strategy to improve therapy in GISTs with acquired or de novo imatinib resistance. Our data illustrate that the inhibition of BRD4 has a robust growth inhibitory effect on GIST cells in vitro, suggesting that secondary imatinib resistance is a result of PI3K/AKT signalling pathway activation.

In imatinib‐resistant GIST cells and xenografts, we observed significant synergistic efficacy with JQ1/imatinib combination therapy. Exposure to JQ1 alone or the JQ1/imatinib combination led to abrogation of AKT activity concomitant with increased caspase activity in cell lines and an increased TUNEL‐labelling index in xenografts. In this context, JQ1‐induced apoptosis in GIST cells is potentially linked to AKT inactivation, given that BRD4 inhibition may perturb the membranous lipid rafts needed for the proper subcellular localization of proteins involved in signal transduction, including proteins in the antiapoptotic PI3K/AKT/mTOR pathway. Moreover, transfection of JQ1‐treated GIST cells with myristoylated AKT counteracted the activities of caspases 3 and 9 and reactivated not only downstream mTOR.

Imatinib, which binds directly to the KIT receptor, was shown to have no appreciable effect on the KIT promoter.^36^ BRD4 inhibition resulting from combined therapy minimized KIT dependency and improved the sensitivity to imatinib in resistant GIST cells in vitro and in vivo.

Accumulating evidence indicates that apoptosis and autophagy share common regulatory elements within oncogenic pathways activated by RTKs, including the PI3K/AKT/mTOR pathway.^42^, ^43^ To date, few studies have described the autophagy‐modulating role of BRD4 inhibition in tumour cells.^44^ One such study reported that glioma cells treated with JQ1 exhibited increased autophagic activity.^45^, ^46^, ^47^ In imatinib‐resistant GIST cells, increased expression of LC3‐II, a widely accepted autophagic marker, was inducible by JQ1, and its level was even higher upon combined treatment with JQ1 and imatinib. Although AKT‐inactivation‐induced apoptosis directs cells to programmed death, the significance of BRD4‐inhibition‐induced autophagy in GIST requires further elucidation. Specifically, the relevant issues regarding JQ1‐induced autophagy include its potential dependency on AKT, engagement of KIT expression and activation and the resultant cytoprotection to maintain energy homeostasis versus cytotoxicity to promote cellular death under nutrient deprivation.^48^ A better understanding of these aspects will help decipher the possible benefits or detriments of autophagic inhibition in GIST cell growth in combined therapy against BRD4 and KIT.

In summary, we have characterized the clinical relevance and biological implications of BRD4, as well as the potential of dual blockade of BRD4 and KIT in GIST. With BRD4 mRNA being preferentially upregulated in aggressive GIST, overexpressed BRD4 confers growth‐promoting and promigratory oncometabolic phenotypes that are associated with adverse clinicopathologic factors and unfavourable RTK genotypes and independently portends decreased DFS. Biologically, an interdependent positive feedback loop may exist to coordinately regulate BRD4, KIT elicited signalling output and the PI3K/AKT/mTOR pathway in GIST. JQ1 represses KIT transactivation to exhaust KIT protein, induces apoptosis and autophagy and inactivates signalling kinases downstream of PI3K/AKT. Therefore, BRD4 inhibition improves the sensitivity to imatinib in resistant cell models and underpins the in vitro and in vivo synergy of JQ1/imatinib combination therapy. Based on the suppressive effects that JQ1 demonstrated, our study provides a rationale for future investigation of novel BRD4 inhibitors with a better therapeutic index in combination with imatinib in GISTs that acquire refractory mutations.

## CONFLICT OF INTEREST

The authors declare no conflict of interest.

## AUTHOR CONTRIBUTIONS

JFM and PDS designed the research study. JFM, PFS and ZMM performed the experiments. JFM analysed the data. JFM and PDS wrote the manuscript. All authors reviewed and approved the manuscript.

## Data Availability

The data used to support the findings of this study are available from the corresponding author upon request.
